# 2,4-Di-*tert*-butyl-6-({[2-(di­methyl­amino)­eth­yl](2-hy­droxy­benz­yl)amino}­meth­yl)phenol

**DOI:** 10.1107/S1600536814010241

**Published:** 2014-05-17

**Authors:** Grzegorz P. Spaleniak, Elwira Bisz, Marzena Białek, Bartosz Zarychta

**Affiliations:** a Faculty of Chemistry, University of Opole, Oleska 48, 45-052 Opole, Poland

## Abstract

The title compound, C_26_H_40_N_2_O_2_, has both its N atoms in trigonal-pyramidal geometries. The mol­ecular structure is stabilized by O—H⋯N and C—H⋯O hydrogen bonds. In the crystal, C—H⋯π inter­actions lead to the formation of a supramolecular helical chain along the *b*-axis direction.

## Related literature   

For general background to the use of di­amine­bis­(aryl­oxido) compounds as tetra­dentate ligands, see: Hirotsu *et al.* (1997 ▶, 1998 ▶); Dutta *et al.* (2011 ▶). For related structures, see: Abrahams *et al.* (2009 ▶); Maity *et al.* (2006 ▶); Janas *et al.* (2012 ▶).
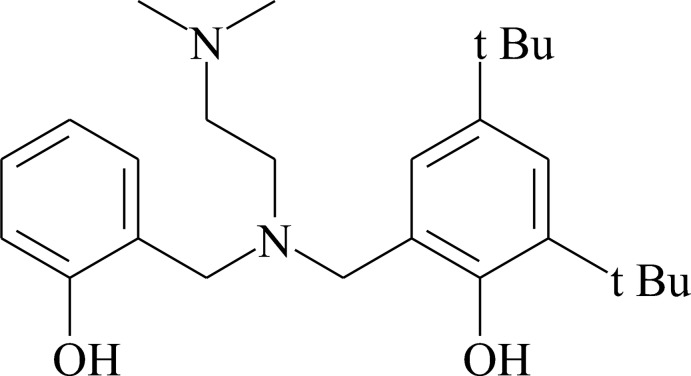


## Experimental   

### 

#### Crystal data  

C_26_H_40_N_2_O_2_
*M* *_r_* = 412.60
Monoclinic, 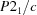

*a* = 12.3002 (7) Å
*b* = 13.3758 (7) Å
*c* = 15.5662 (9) Å
β = 96.377 (5)°
*V* = 2545.2 (2) Å^3^
*Z* = 4
Mo *K*α radiation
μ = 0.07 mm^−1^
*T* = 150 K
0.40 × 0.37 × 0.35 mm

#### Data collection  

Oxford Diffraction Xcalibur diffractometer
17015 measured reflections
4964 independent reflections
2292 reflections with *I* > 2σ(*I*)
*R*_int_ = 0.047

#### Refinement  

*R*[*F* ^2^ > 2σ(*F* ^2^)] = 0.044
*wR*(*F* ^2^) = 0.099
*S* = 0.78
4964 reflections
277 parameters
H-atom parameters constrained
Δρ_max_ = 0.22 e Å^−3^
Δρ_min_ = −0.18 e Å^−3^

### 

Data collection: *CrysAlis CCD* (Oxford Diffraction, 2008 ▶); cell refinement: *CrysAlis RED* (Oxford Diffraction, 2008 ▶); data reduction: *CrysAlis RED*; program(s) used to solve structure: *SHELXS2013* (Sheldrick, 2008 ▶); program(s) used to refine structure: *SHELXL2013* (Sheldrick, 2008 ▶); molecular graphics: *SHELXTL* (Sheldrick, 2008 ▶); software used to prepare material for publication: *SHELXTL*.

## Supplementary Material

Crystal structure: contains datablock(s) global, I. DOI: 10.1107/S1600536814010241/bt6979sup1.cif

Structure factors: contains datablock(s) I. DOI: 10.1107/S1600536814010241/bt6979Isup2.hkl

Supporting information file. DOI: 10.1107/S1600536814010241/bt6979Isup3.cml

CCDC reference: 1001222

Additional supporting information:  crystallographic information; 3D view; checkCIF report

## Figures and Tables

**Table 1:** Hydrogen-bond geometry (Å, °) *Cg* is the centroid of the C13–C18 ring.

*D*—H⋯*A*	*D*—H	H⋯*A*	*D*⋯*A*	*D*—H⋯*A*
O1—H1*A*⋯N1	0.96 (2)	2.59 (2)	3.1610 (19)	118.5 (16)
O1—H1*A*⋯N2	0.96 (2)	1.89 (2)	2.824 (2)	162.4 (19)
O2—H2*A*⋯N1	0.88 (2)	1.95 (2)	2.7563 (18)	152.1 (18)
C3—H3*A*⋯O2	0.96	2.64	3.411 (3)	137
C9—H9*A*⋯*Cg* ^i^	0.93	2.77	3.593 (2)	148

Symmetry code: (i) 
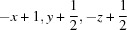
.

**Fig. 1.**
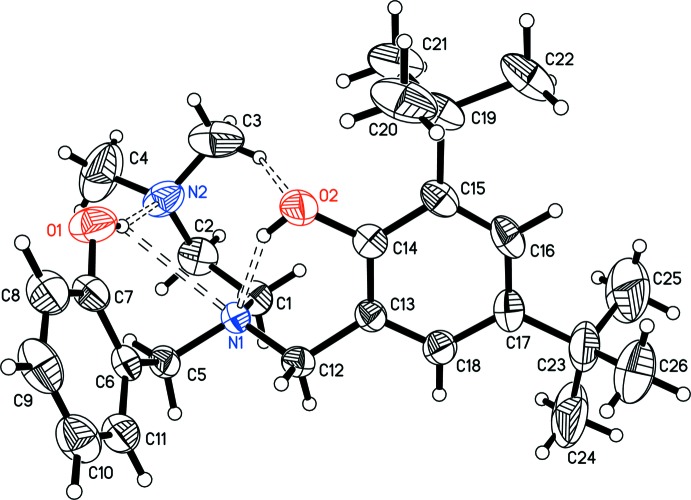
The molecular structure of the title compound showing 50% displacement ellipsoids (arbitrary spheres for the H atoms).

**Fig. 2.**
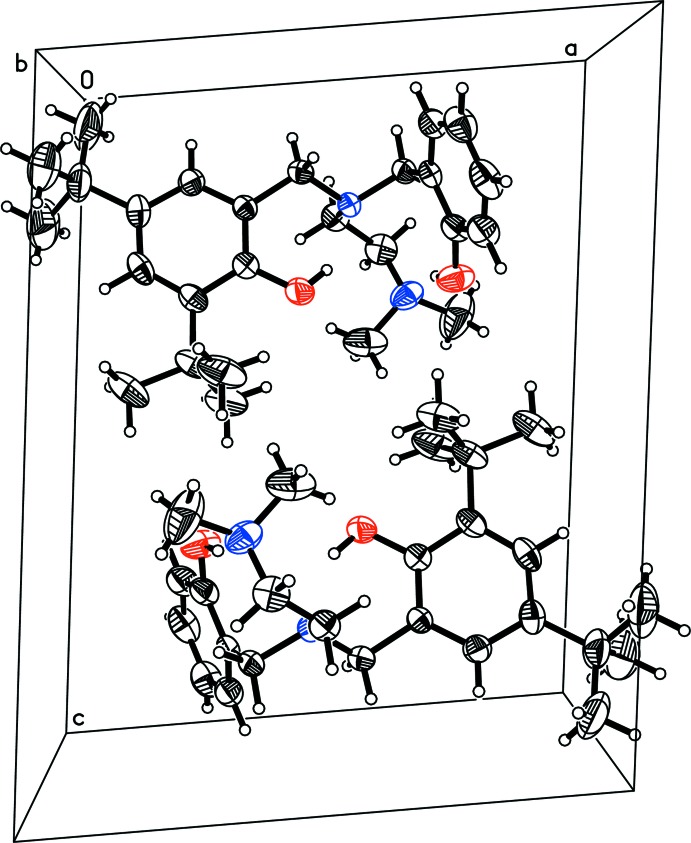
A packing diagram of the title compound.

## supplementary crystallographic information

### 1. Comment

Diamine bis(phenolate) compounds and its derivatives represent the dominant class of compounds that are used as tripodal tetradentate ligands with an N_2_O_2_ donor set (Hirotsu *et al.*, 1997, 1998). The steric factors of substituents in the tetradentate ligands are especially important in complexation of agents for polymerization reactions.

The molecular structure of the title compound and the atom-numbering scheme are shown in Figure 1. The crystal structure shows trigonal pyramidal geometries around N1 [sum of C—N—C angles = 332.05 (18)° and N2 [sum of C—N—C angles = 330.37 (13)°], and is comparable with related structures (Abrahams *et al.*, 2009; Maity *et al.*, 2006; Janas *et al.*, 2012). The aromatic ring which is substituted with *tert*-butyl groups is slightly more distorted in comparison to unsubstituted one due to the steric hindrance. The molecular structure is stabilized by three hydrogen bonds between hydroxyl groups and amine nitrogen atoms (Figure 1) and one of a C—H···O type. This pattern of interactions influences the dihedral angle between aromatic moieties which amounts 73.88 (57)°. Such stabilization is also observed for structures when both aromatic rings are substituted (Maity *et al.*, 2006; Janas *et al.*, 2012), in contrast to the unsubstituted molecule (Abrahams *et al.*, 2009). All remaining bond distances and angles are normal and in good agreement with the geometries of other diamine bis(phenolates) (Abrahams *et al.*, 2009; Maity *et al.*, 2006; Janas *et al.*, 2012). Strong intermolecular interactions were not found in the crystal. The structure is stabilized by weak C—H···π interactions.

### 2. Experimental

The compound was prepared according to literature procedure (Dutta *et al.*, 2011). Crystals suitable for X-ray crystal structure analysis were grown from methanol.

### 3. Refinement

H atoms bonded to C were positioned geometrically and treated as riding on their parent atoms with C—H = 0.93 - 0.97 Å and with *U*_iso_(H) = 1.5*U*_eq_ (C-methyl) or = 1.2U _eq_(C) for other H atoms. The coordinates of the H atoms bonded to O were refined with *U*_iso_(H) = 1.5*U*_eq_(O).

### Crystal data

**Table:**

C_26_H_40_N_2_O_2_	*F*(000) = 904
*M**_r_* = 412.60	*D*_x_ = 1.077 Mg m^−^^3^
Monoclinic, *P*2_1_/*c*	Mo *K*α radiation, λ = 0.71073 Å
*a* = 12.3002 (7) Å	Cell parameters from 17015 reflections
*b* = 13.3758 (7) Å	θ = 3.0–26.0°
*c* = 15.5662 (9) Å	µ = 0.07 mm^−^^1^
β = 96.377 (5)°	*T* = 150 K
*V* = 2545.2 (2) Å^3^	Cubic, colourless
*Z* = 4	0.4 × 0.37 × 0.35 mm

### Data collection

**Table:**

Oxford Diffraction Xcalibur diffractometer	2292 reflections with *I* > 2σ(*I*)
Radiation source: Enhance (Mo) X-ray Source	*R*_int_ = 0.047
Graphite monochromator	θ_max_ = 26.0°, θ_min_ = 3.0°
Detector resolution: 10.4508 pixels mm^-1^	*h* = −15→15
ω–scan	*k* = −16→9
17015 measured reflections	*l* = −19→19
4964 independent reflections	

### Refinement

**Table:**

Refinement on *F*^2^	0 restraints
Least-squares matrix: full	Hydrogen site location: mixed
*R*[*F*^2^ > 2σ(*F*^2^)] = 0.044	H-atom parameters constrained
*wR*(*F*^2^) = 0.099	*w* = 1/[σ^2^(*F*_o_^2^) + (0.0474*P*)^2^] where *P* = (*F*_o_^2^ + 2*F*_c_^2^)/3
*S* = 0.78	(Δ/σ)_max_ < 0.001
4964 reflections	Δρ_max_ = 0.22 e Å^−^^3^
277 parameters	Δρ_min_ = −0.18 e Å^−^^3^

### Special details

**Table:**

Geometry. All e.s.d.'s (except the e.s.d. in the dihedral angle between two l.s. planes) are estimated using the full covariance matrix. The cell e.s.d.'s are taken into account individually in the estimation of e.s.d.'s in distances, angles and torsion angles; correlations between e.s.d.'s in cell parameters are only used when they are defined by crystal symmetry. An approximate (isotropic) treatment of cell e.s.d.'s is used for estimating e.s.d.'s involving l.s. planes.

### Fractional atomic coordinates and isotropic or equivalent isotropic displacement parameters (Å^2^)

**Table:**

	*x*	*y*	*z*	*U*_iso_*/*U*_eq_	
N1	0.47272 (11)	0.82096 (10)	0.29297 (9)	0.0297 (4)	
N2	0.35360 (14)	0.71832 (12)	0.15196 (11)	0.0516 (5)	
O1	0.27532 (12)	0.91451 (11)	0.17200 (8)	0.0573 (4)	
H1A	0.3077 (17)	0.8489 (17)	0.1774 (13)	0.086*	
O2	0.56148 (11)	0.93511 (9)	0.17087 (8)	0.0449 (4)	
H2A	0.5129 (16)	0.9043 (14)	0.1990 (13)	0.067*	
C1	0.49556 (15)	0.71454 (12)	0.27880 (12)	0.0409 (5)	
H1B	0.5097	0.6816	0.3344	0.049*	
H1C	0.5613	0.7092	0.2500	0.049*	
C2	0.40348 (16)	0.66064 (14)	0.22545 (13)	0.0501 (6)	
H2B	0.4314	0.5985	0.2044	0.060*	
H2C	0.3475	0.6439	0.2623	0.060*	
C3	0.4308 (2)	0.72638 (16)	0.08655 (14)	0.0735 (7)	
H3A	0.4974	0.7570	0.1119	0.110*	
H3B	0.3990	0.7666	0.0392	0.110*	
H3C	0.4464	0.6609	0.0659	0.110*	
C4	0.2528 (2)	0.67198 (19)	0.11449 (18)	0.0988 (9)	
H4A	0.2046	0.7223	0.0880	0.148*	
H4B	0.2183	0.6388	0.1590	0.148*	
H4C	0.2687	0.6241	0.0717	0.148*	
C5	0.37250 (14)	0.83566 (13)	0.33576 (11)	0.0344 (5)	
H5A	0.3883	0.8212	0.3969	0.041*	
H5B	0.3167	0.7892	0.3117	0.041*	
C6	0.32984 (14)	0.94016 (13)	0.32456 (12)	0.0331 (4)	
C7	0.28295 (15)	0.97286 (14)	0.24381 (13)	0.0405 (5)	
C8	0.23990 (16)	1.06898 (15)	0.23475 (14)	0.0508 (5)	
H8A	0.2085	1.0906	0.1808	0.061*	
C9	0.24319 (17)	1.13236 (15)	0.30464 (16)	0.0551 (6)	
H9A	0.2142	1.1964	0.2977	0.066*	
C10	0.28917 (17)	1.10118 (16)	0.38444 (15)	0.0547 (6)	
H10A	0.2914	1.1437	0.4319	0.066*	
C11	0.33222 (15)	1.00577 (14)	0.39350 (12)	0.0443 (5)	
H11A	0.3638	0.9850	0.4476	0.053*	
C12	0.56595 (14)	0.86832 (13)	0.34509 (11)	0.0351 (5)	
H12A	0.5835	0.8298	0.3976	0.042*	
H12B	0.5444	0.9347	0.3617	0.042*	
C13	0.66716 (14)	0.87681 (12)	0.29899 (11)	0.0320 (4)	
C14	0.66188 (15)	0.91250 (12)	0.21458 (11)	0.0336 (4)	
C15	0.75646 (16)	0.92661 (12)	0.17339 (12)	0.0377 (5)	
C16	0.85514 (16)	0.90452 (13)	0.22224 (13)	0.0457 (5)	
H16A	0.9192	0.9150	0.1969	0.055*	
C17	0.86462 (15)	0.86777 (14)	0.30655 (14)	0.0453 (5)	
C18	0.76839 (15)	0.85408 (13)	0.34324 (12)	0.0398 (5)	
H18A	0.7716	0.8289	0.3992	0.048*	
C19	0.75220 (17)	0.96199 (14)	0.07887 (12)	0.0477 (5)	
C20	0.6966 (2)	1.06485 (15)	0.06686 (14)	0.0759 (7)	
H20A	0.6236	1.0608	0.0830	0.114*	
H20B	0.6937	1.0848	0.0074	0.114*	
H20C	0.7376	1.1132	0.1027	0.114*	
C21	0.69022 (19)	0.88603 (16)	0.01895 (13)	0.0700 (7)	
H21A	0.6177	0.8777	0.0351	0.105*	
H21B	0.7279	0.8231	0.0237	0.105*	
H21C	0.6861	0.9095	−0.0396	0.105*	
C22	0.86723 (18)	0.97404 (17)	0.04988 (15)	0.0780 (8)	
H22A	0.8609	0.9961	−0.0091	0.117*	
H22B	0.9047	0.9110	0.0548	0.117*	
H22C	0.9077	1.0225	0.0860	0.117*	
C23	0.97742 (17)	0.84687 (19)	0.35613 (16)	0.0669 (7)	
C24	0.9681 (2)	0.7884 (2)	0.43981 (19)	0.1249 (13)	
H24A	0.9248	0.8258	0.4762	0.187*	
H24B	1.0398	0.7778	0.4695	0.187*	
H24C	0.9339	0.7250	0.4261	0.187*	
C25	1.0477 (2)	0.7853 (2)	0.3005 (2)	0.1166 (11)	
H25A	1.0562	0.8211	0.2482	0.175*	
H25B	1.0128	0.7223	0.2865	0.175*	
H25C	1.1183	0.7739	0.3319	0.175*	
C26	1.0338 (2)	0.9463 (2)	0.37872 (19)	0.1113 (10)	
H26A	1.0413	0.9828	0.3266	0.167*	
H26B	1.1048	0.9343	0.4091	0.167*	
H26C	0.9907	0.9845	0.4147	0.167*	

### Atomic displacement parameters (Å^2^)

**Table:**

	*U*^11^	*U*^22^	*U*^33^	*U*^12^	*U*^13^	*U*^23^
N1	0.0285 (9)	0.0291 (8)	0.0319 (9)	−0.0003 (7)	0.0056 (7)	0.0007 (7)
N2	0.0611 (12)	0.0471 (10)	0.0436 (11)	0.0080 (10)	−0.0073 (9)	−0.0106 (9)
O1	0.0767 (11)	0.0558 (9)	0.0378 (8)	0.0229 (8)	−0.0003 (7)	−0.0001 (8)
O2	0.0436 (9)	0.0540 (9)	0.0377 (8)	0.0040 (7)	0.0079 (6)	0.0137 (7)
C1	0.0445 (12)	0.0346 (11)	0.0439 (12)	0.0056 (10)	0.0068 (10)	0.0058 (9)
C2	0.0594 (15)	0.0370 (12)	0.0540 (14)	−0.0013 (11)	0.0073 (11)	−0.0051 (11)
C3	0.112 (2)	0.0638 (15)	0.0458 (14)	0.0141 (15)	0.0120 (14)	−0.0037 (12)
C4	0.0679 (19)	0.097 (2)	0.122 (2)	0.0002 (16)	−0.0302 (17)	−0.0425 (18)
C5	0.0319 (11)	0.0385 (11)	0.0336 (11)	−0.0021 (9)	0.0074 (9)	0.0053 (9)
C6	0.0273 (10)	0.0359 (11)	0.0378 (11)	−0.0006 (9)	0.0109 (8)	0.0010 (10)
C7	0.0424 (13)	0.0417 (12)	0.0394 (12)	0.0049 (10)	0.0133 (10)	0.0010 (10)
C8	0.0541 (14)	0.0483 (13)	0.0519 (14)	0.0123 (11)	0.0143 (11)	0.0161 (12)
C9	0.0554 (15)	0.0361 (12)	0.0785 (18)	0.0055 (11)	0.0285 (13)	0.0044 (13)
C10	0.0543 (15)	0.0457 (14)	0.0667 (17)	−0.0022 (11)	0.0182 (12)	−0.0141 (12)
C11	0.0410 (13)	0.0488 (13)	0.0442 (13)	−0.0008 (10)	0.0091 (10)	−0.0020 (11)
C12	0.0358 (12)	0.0401 (11)	0.0297 (11)	0.0012 (9)	0.0061 (9)	0.0030 (9)
C13	0.0319 (12)	0.0312 (10)	0.0334 (11)	−0.0018 (9)	0.0064 (9)	0.0001 (9)
C14	0.0366 (12)	0.0285 (10)	0.0362 (11)	0.0013 (9)	0.0062 (9)	0.0012 (9)
C15	0.0451 (13)	0.0280 (10)	0.0428 (12)	−0.0041 (9)	0.0169 (10)	0.0001 (9)
C16	0.0397 (13)	0.0431 (12)	0.0580 (14)	−0.0096 (10)	0.0220 (11)	0.0009 (11)
C17	0.0313 (13)	0.0474 (12)	0.0576 (14)	−0.0058 (10)	0.0066 (11)	0.0001 (11)
C18	0.0379 (13)	0.0438 (12)	0.0376 (12)	−0.0071 (10)	0.0038 (10)	0.0032 (9)
C19	0.0650 (15)	0.0387 (12)	0.0440 (12)	−0.0021 (11)	0.0267 (11)	0.0042 (10)
C20	0.121 (2)	0.0540 (14)	0.0595 (15)	0.0186 (14)	0.0394 (14)	0.0210 (12)
C21	0.098 (2)	0.0701 (16)	0.0453 (14)	−0.0130 (14)	0.0229 (13)	−0.0015 (12)
C22	0.0887 (19)	0.0829 (17)	0.0714 (16)	−0.0115 (15)	0.0486 (14)	0.0091 (14)
C23	0.0317 (14)	0.0890 (19)	0.0786 (18)	−0.0103 (13)	−0.0003 (12)	0.0107 (15)
C24	0.0437 (16)	0.203 (4)	0.121 (2)	−0.0086 (19)	−0.0222 (16)	0.078 (2)
C25	0.0521 (17)	0.152 (3)	0.144 (3)	0.0380 (18)	0.0031 (18)	−0.006 (2)
C26	0.0636 (19)	0.146 (3)	0.121 (2)	−0.0468 (18)	−0.0043 (17)	−0.016 (2)

### Geometric parameters (Å, º)

**Table:**

N1—C12	1.472 (2)	C12—H12B	0.9700
N1—C1	1.472 (2)	C13—C18	1.388 (2)
N1—C5	1.478 (2)	C13—C14	1.393 (2)
N2—C4	1.450 (2)	C14—C15	1.402 (2)
N2—C2	1.457 (2)	C15—C16	1.391 (2)
N2—C3	1.471 (2)	C15—C19	1.541 (2)
O1—C7	1.358 (2)	C16—C17	1.394 (2)
O1—H1A	0.96 (2)	C16—H16A	0.9300
O2—C14	1.376 (2)	C17—C18	1.382 (2)
O2—H2A	0.88 (2)	C17—C23	1.537 (3)
C1—C2	1.511 (2)	C18—H18A	0.9300
C1—H1B	0.9700	C19—C21	1.525 (3)
C1—H1C	0.9700	C19—C20	1.539 (3)
C2—H2B	0.9700	C19—C22	1.541 (3)
C2—H2C	0.9700	C20—H20A	0.9600
C3—H3A	0.9600	C20—H20B	0.9600
C3—H3B	0.9600	C20—H20C	0.9600
C3—H3C	0.9600	C21—H21A	0.9600
C4—H4A	0.9600	C21—H21B	0.9600
C4—H4B	0.9600	C21—H21C	0.9600
C4—H4C	0.9600	C22—H22A	0.9600
C5—C6	1.496 (2)	C22—H22B	0.9600
C5—H5A	0.9700	C22—H22C	0.9600
C5—H5B	0.9700	C23—C26	1.523 (3)
C6—C11	1.384 (2)	C23—C25	1.530 (3)
C6—C7	1.394 (2)	C23—C24	1.535 (3)
C7—C8	1.392 (2)	C24—H24A	0.9600
C8—C9	1.376 (3)	C24—H24B	0.9600
C8—H8A	0.9300	C24—H24C	0.9600
C9—C10	1.371 (3)	C25—H25A	0.9600
C9—H9A	0.9300	C25—H25B	0.9600
C10—C11	1.383 (3)	C25—H25C	0.9600
C10—H10A	0.9300	C26—H26A	0.9600
C11—H11A	0.9300	C26—H26B	0.9600
C12—C13	1.509 (2)	C26—H26C	0.9600
C12—H12A	0.9700		
			
C12—N1—C1	110.41 (13)	C14—C13—C12	121.34 (16)
C12—N1—C5	109.39 (13)	O2—C14—C13	119.15 (16)
C1—N1—C5	112.25 (13)	O2—C14—C15	119.28 (16)
C4—N2—C2	110.86 (18)	C13—C14—C15	121.57 (17)
C4—N2—C3	109.99 (18)	C16—C15—C14	116.07 (17)
C2—N2—C3	109.52 (17)	C16—C15—C19	121.50 (17)
C7—O1—H1A	117.5 (13)	C14—C15—C19	122.41 (18)
C14—O2—H2A	105.8 (13)	C15—C16—C17	124.50 (17)
N1—C1—C2	113.57 (14)	C15—C16—H16A	117.8
N1—C1—H1B	108.9	C17—C16—H16A	117.8
C2—C1—H1B	108.9	C18—C17—C16	116.72 (18)
N1—C1—H1C	108.9	C18—C17—C23	122.4 (2)
C2—C1—H1C	108.9	C16—C17—C23	120.89 (18)
H1B—C1—H1C	107.7	C17—C18—C13	121.88 (18)
N2—C2—C1	113.78 (15)	C17—C18—H18A	119.1
N2—C2—H2B	108.8	C13—C18—H18A	119.1
C1—C2—H2B	108.8	C21—C19—C20	109.63 (19)
N2—C2—H2C	108.8	C21—C19—C22	107.67 (17)
C1—C2—H2C	108.8	C20—C19—C22	106.35 (16)
H2B—C2—H2C	107.7	C21—C19—C15	109.94 (15)
N2—C3—H3A	109.5	C20—C19—C15	110.98 (15)
N2—C3—H3B	109.5	C22—C19—C15	112.14 (18)
H3A—C3—H3B	109.5	C19—C20—H20A	109.5
N2—C3—H3C	109.5	C19—C20—H20B	109.5
H3A—C3—H3C	109.5	H20A—C20—H20B	109.5
H3B—C3—H3C	109.5	C19—C20—H20C	109.5
N2—C4—H4A	109.5	H20A—C20—H20C	109.5
N2—C4—H4B	109.5	H20B—C20—H20C	109.5
H4A—C4—H4B	109.5	C19—C21—H21A	109.5
N2—C4—H4C	109.5	C19—C21—H21B	109.5
H4A—C4—H4C	109.5	H21A—C21—H21B	109.5
H4B—C4—H4C	109.5	C19—C21—H21C	109.5
N1—C5—C6	111.78 (13)	H21A—C21—H21C	109.5
N1—C5—H5A	109.3	H21B—C21—H21C	109.5
C6—C5—H5A	109.3	C19—C22—H22A	109.5
N1—C5—H5B	109.3	C19—C22—H22B	109.5
C6—C5—H5B	109.3	H22A—C22—H22B	109.5
H5A—C5—H5B	107.9	C19—C22—H22C	109.5
C11—C6—C7	118.12 (17)	H22A—C22—H22C	109.5
C11—C6—C5	121.66 (17)	H22B—C22—H22C	109.5
C7—C6—C5	120.18 (16)	C26—C23—C25	109.2 (2)
O1—C7—C8	117.18 (18)	C26—C23—C24	108.9 (2)
O1—C7—C6	123.08 (16)	C25—C23—C24	107.8 (2)
C8—C7—C6	119.73 (18)	C26—C23—C17	108.6 (2)
C9—C8—C7	120.8 (2)	C25—C23—C17	110.4 (2)
C9—C8—H8A	119.6	C24—C23—C17	111.77 (18)
C7—C8—H8A	119.6	C23—C24—H24A	109.5
C10—C9—C8	120.11 (19)	C23—C24—H24B	109.5
C10—C9—H9A	119.9	H24A—C24—H24B	109.5
C8—C9—H9A	119.9	C23—C24—H24C	109.5
C9—C10—C11	119.2 (2)	H24A—C24—H24C	109.5
C9—C10—H10A	120.4	H24B—C24—H24C	109.5
C11—C10—H10A	120.4	C23—C25—H25A	109.5
C10—C11—C6	122.08 (19)	C23—C25—H25B	109.5
C10—C11—H11A	119.0	H25A—C25—H25B	109.5
C6—C11—H11A	119.0	C23—C25—H25C	109.5
N1—C12—C13	113.81 (14)	H25A—C25—H25C	109.5
N1—C12—H12A	108.8	H25B—C25—H25C	109.5
C13—C12—H12A	108.8	C23—C26—H26A	109.5
N1—C12—H12B	108.8	C23—C26—H26B	109.5
C13—C12—H12B	108.8	H26A—C26—H26B	109.5
H12A—C12—H12B	107.7	C23—C26—H26C	109.5
C18—C13—C14	119.23 (16)	H26A—C26—H26C	109.5
C18—C13—C12	119.32 (16)	H26B—C26—H26C	109.5
			
C12—N1—C1—C2	−179.85 (14)	C18—C13—C14—C15	−0.4 (2)
C5—N1—C1—C2	57.8 (2)	C12—C13—C14—C15	175.84 (16)
C4—N2—C2—C1	−168.19 (17)	O2—C14—C15—C16	178.47 (15)
C3—N2—C2—C1	70.3 (2)	C13—C14—C15—C16	−1.1 (2)
N1—C1—C2—N2	41.7 (2)	O2—C14—C15—C19	−3.0 (2)
C12—N1—C5—C6	75.50 (18)	C13—C14—C15—C19	177.50 (16)
C1—N1—C5—C6	−161.58 (14)	C14—C15—C16—C17	1.7 (3)
N1—C5—C6—C11	−111.69 (18)	C19—C15—C16—C17	−176.85 (17)
N1—C5—C6—C7	70.4 (2)	C15—C16—C17—C18	−0.8 (3)
C11—C6—C7—O1	−179.36 (16)	C15—C16—C17—C23	−179.12 (18)
C5—C6—C7—O1	−1.4 (3)	C16—C17—C18—C13	−0.7 (3)
C11—C6—C7—C8	−0.3 (3)	C23—C17—C18—C13	177.50 (18)
C5—C6—C7—C8	177.65 (16)	C14—C13—C18—C17	1.3 (3)
O1—C7—C8—C9	179.21 (17)	C12—C13—C18—C17	−174.96 (16)
C6—C7—C8—C9	0.1 (3)	C16—C15—C19—C21	116.8 (2)
C7—C8—C9—C10	0.0 (3)	C14—C15—C19—C21	−61.7 (2)
C8—C9—C10—C11	0.2 (3)	C16—C15—C19—C20	−121.7 (2)
C9—C10—C11—C6	−0.4 (3)	C14—C15—C19—C20	59.8 (2)
C7—C6—C11—C10	0.5 (3)	C16—C15—C19—C22	−2.9 (2)
C5—C6—C11—C10	−177.45 (16)	C14—C15—C19—C22	178.58 (17)
C1—N1—C12—C13	68.05 (18)	C18—C17—C23—C26	−107.3 (2)
C5—N1—C12—C13	−167.94 (13)	C16—C17—C23—C26	70.9 (3)
N1—C12—C13—C18	−136.89 (16)	C18—C17—C23—C25	132.9 (2)
N1—C12—C13—C14	46.9 (2)	C16—C17—C23—C25	−48.9 (3)
C18—C13—C14—O2	−179.92 (15)	C18—C17—C23—C24	12.9 (3)
C12—C13—C14—O2	−3.7 (2)	C16—C17—C23—C24	−168.9 (2)

### Hydrogen-bond geometry (Å, º)

Cg is the centroid of the C13–C18 ring.

**Table:**

*D*—H···*A*	*D*—H	H···*A*	*D*···*A*	*D*—H···*A*
O1—H1*A*···N1	0.96 (2)	2.59 (2)	3.1610 (19)	118.5 (16)
O1—H1*A*···N2	0.96 (2)	1.89 (2)	2.824 (2)	162.4 (19)
O2—H2*A*···N1	0.88 (2)	1.95 (2)	2.7563 (18)	152.1 (18)
C3—H3*A*···O2	0.96	2.64	3.411 (3)	137
C9—H9*A*···*Cg*^i^	0.93	2.77	3.593 (2)	148

Symmetry code: (i) −*x*+1, *y*+1/2, −*z*+1/2.
